# Stress and Disease Onset in Antineutrophil Cytoplasmic Antibody-Associated Vasculitis

**DOI:** 10.3389/fpsyt.2017.00286

**Published:** 2017-12-15

**Authors:** Christina V. Golemati, Clio P. Mavragani, Sophia Lionaki, Dimitrios Karaiskos, Haralampos M. Moutsopoulos

**Affiliations:** ^1^Department of Pathophysiology, Faculty of Medicine, National and Kapodistrian University of Athens, Athens, Greece; ^2^Department of Physiology, Faculty of Medicine, National and Kapodistrian University of Athens, Athens, Greece; ^3^Department of Nephrology, Laiko Hospital, National and Kapodistrian University of Athens, Athens, Greece

**Keywords:** ANCA-associated vasculitis, stressful events, social support, coping, depression, anxiety

## Abstract

**Objective:**

To explore the potential contribution of stress as a trigger for disease onset in patients with antineutrophil cytoplasmic antibody (ANCA) associated vasculitis (AAV).

**Methods:**

53 AAV and 85 rheumatoid arthritis (RA) patients as well as 53 healthy controls (HC) were thoroughly asked for the number and impact of stressful life events, coping strategies, and available social support 12 months prior to disease onset. Anxiety, depression, personality dimensions, insomnia, and fatigue were also determined.

**Results:**

AAV patients reported higher scoring of the impact of stressful life events compared to the RA and HC group prior to disease onset (2.8 ± 3.1 vs 1.8 ± 2.1 vs 1.7 ± 2.3, *p*-values: 0.047 and 0.053, respectively). While the number of reported stressful events was found to be significantly higher in AAV vs RA patients but not HC, certain coping strategies and social support features were more commonly implemented by AAV patients compared to HC, but not RA patients. As far as personality and other psychosocial characteristics, AAV patients displayed significantly higher psychoticism traits compared to RA, with no other differences being detected between AAV patients and both RA and HC. After adjusting for potential cofounders, scoring of the impact of stressful life events >3 was independently associated with AAV development compared to both RA and HC [ORs (95% CI): 4.6 (1.6–13.4) and 4.4 (1.0–19.0), respectively].

**Conclusion:**

The perceived impact of stressful life events prior to disease onset emerged as a contributing factor for AAV development.

## Significance and Innovations

- This is the first report regarding stress and AAV development.
- The perceived degree of negative impact of stressful life events was found higher for AAV patients and independently associated with AAV status even after adjusting for potential confounding psychosocial variables.

## Introduction

Antineutrophil cytoplasmic antibody (ANCA)-associated vasculitis (AAV) is a group of rare clinical conditions affecting mainly middle aged individuals, characterized by vascular destruction and tissue necrosis (1–3). In the presence of major organ involvement, these chronic multisystem disorders may be life-threatening and require intense immunosuppression and long-term maintenance treatment (4).

It is widely accepted that genetic, environmental, and hormonal contributors are implicated in the development of autoimmune diseases (5) with emotional and physiological sensitivity to stress, less coping resources and imbalanced immune responses being important contributors. Stress has been previously emerged as a critical determinant of disease onset in autoimmune populations, including Grave’s disease, Sjogren’s syndrome (SS), and childhood arthritis (6–9). Increased proinflammatory soluble mediators such as interleukin (IL)-1, IL-6, and Th17 related cytokines, imbalance between Th1 and Th2 responses, as well as defective function of T regulatory cells have been all postulated as potential mechanisms (10). Mounting evidence also link childhood adversities with immune dysregulation in adult life (11). Children exposed to high stress in families showed enhanced immune responses against diabetes associated autoantigens in relation to controls (12).

According to Lazarus theory, stress is defined as an imbalance between environmental demands and an individual’s capacity to adapt to these demands (13). Stress is usually experienced as a negative state (14) and is perceived as the response to a stressor. Stressors can be classified as dispositional or environmental, physical (such as infection), or psychological as well as acute or chronic. Depending their type and duration, they may lead to neuroendocrine and immune alterations (14). While stressors or triggers of stress lead to the activation of physiological systems designed to ensure survival and protect the organism, in the presence of an excess of the bodily reactions, diseases can develop (15). Stressful life events are a type of stressors, which are “discrete, observable events standing for significant life changes and possessing a relatively clear onset and offset” (16). Chronic stress can develop, either as a response to life events, daily stressors, or exposure to trauma. Intense or chronic responses, as a result of life events, can have significant consequences in mental and physical health (17). It should, however, be emphasized that the event itself cannot solely account for the resulting psychological distress; “the soil in which it falls,” hence the impact of the event on each individual, is also of major importance (18).

In an attempt to adapt to stressful life events, with an ultimate goal for better survival, individuals develop coping strategies, which are resources that one has to fight against stress. Depending on the type of coping implemented, two main strategies, namely engagement and disengagement are recognized. The first requires active psychological involvement, while the second is based on a tendency to move away from the source of stress. A great deal of research associates engagement coping with lower psychological distress and better health and disengagement coping with greater distress and worse health outcomes (19, 20). In addition to coping strategies, other psychological variables such as social support, anxiety, depression, personality characteristics in addition to health-related variables such as fatigue and sleep disturbances, are thought to contribute to disease (21, 22).

Increased fatigue, impaired quality of life, and work disability have previously been linked to psychological distress and depression in AAV patients, compared to healthy populations, and thus representing a major disease burden (23–27). Patients with Wegener’s granulomatosis with worse mental health at remission had increased likelihood of disease flares in the immediate future (28). Similarly, distinct psychological and personality characteristics have been earlier reported in several autoimmune diseases including systemic lupus erythematosus (SLE) (29), SS (30, 31) systemic sclerosis (SSc) (32), and rheumatoid arthritis (RA) (33–35). These patients tend to suffer more commonly by depressive and anxiety symptoms compared to controls (31, 32), to receive less social support (8, 32), as well as to implement dysfunctional coping attitudes such as denial, behavioral disengagement and venting of emotions (8, 29, 32). Additionally, trait anxiety, obsessiveness (30, 31), less extraversion, and neuroticism (32) are among the main personality features found to characterize these patient populations and were shown to predict long-term psychological distress (33).

Though negative stressful life events and defective coping strategies have been previously recognized as important triggers in several autoimmune diseases, such as SLE, Sjogren, SSc, RA, data relating psychosocial variables prior to AAV onset are scarce. In this setting, the goal of the current study was to explore whether the number and impact of negative stressful life events could affect the risk for AAV development. Implemented coping strategies, availability of social support resources, as well as personality and psychosocial characteristics were taken into account.

## Materials and Methods

### Study Participants

An inception cohort of 147 biopsy-proven AAV patients described elsewhere (36) was used as a registry source. Of these, 94 individuals were not included in the study, as 13 had died, 25 refused to participate, and 56 had changed contact information and thus, they could not be reached. The final sample consisted of 53 AAV patients, 53 age- and gender-matched healthy controls (HC) and 85 RA patients diagnosed according to the American College of Rheumatology (37) classification criteria with similar timing of disease onset with AAV cohort. Given that responses to various questionnaires are dependent on general socioeconomic situation, available HC data (*n* = 34) from a previous study (conducted in our department with the exact same methodology) have been used (8). Thus, HC and RA patients were also matched to AAV patients for the year of occurrence of the reported stressful life events. Exclusion criteria for HC included the carriage of a diagnosis of any autoimmune disease and severe psychiatric history requiring treatment. This study was carried out in accordance with the recommendations of General Hospital of Athens “Laiko Ethics Committee,” with written informed consent from all subjects in accordance with the Declaration of Helsinki.

### Demographics and Disease Characteristics

A battery of psychometric scales and questionnaires was administered to patients and controls. All participants were asked about their personal, educational, and occupational status, smoking habits, alcohol consumption, and physical activities. Information about disease duration, clinical, and serological characteristics, treatment and past medical history was derived from patients’ medical records. A Birmingham Vasculitis Activity Score (BVAS) (38) was used for AAV patients to assess disease activity at certain times.

### Instruments

#### Stressful Life Events, Coping Strategies, and Social Support

Patients and controls completed the previously validated Greek versions of several questionnaires (39) regarding the occurrence of stressful life events prior to disease onset (premorbid period) and their psychosocial adjustment. Stressful life events were evaluated with Life Experiences Survey (40), a questionnaire that explores the number of major events (such as injury or death of a loved one, changes at work, giving birth, etc.) in the preceding year, and their subjective impact (positive, negative or neutral, subjective perception of psychological stress), assessed as a score resulting from the sum of individual answers in a Likert scale ranging from 0 to 3 (α = 0.77) (41). Only negative events mentioned were evaluated, because of the retrospective methodology of the study. Patients were asked if they had experienced any negative stressful events in the preceding 12 months of their diagnosis. HC were asked about negative stressful events in the preceding 12 months of the interview day. Psychosocial adjustment including the development of coping strategies and the perceived availability of social support resources was evaluated with the *COPE Inventory* (COPE) (42) consisting of 60 items (α = 0.88) and the *Medical Outcomes Study, Social Support Survey* (43) consisting of 19 items (α = 0.94), respectively.

#### Psychological Variables

Additional psychometric variables of personality, state and trait anxiety, depression, insomnia, and fatigue were measured by the following questionnaires: Eysenck Personality Questionnaire (44) consisting of 86 items on a yes/no answer evaluating four personality dimensions: neuroticism (α = 0.88), extraversion (α = 0.83), psychoticism (α = 0.67), and lie (α = 0.67); State-Trait Anxiety Inventory (45) consisting of 40 items, evaluating temporary anxiety (state, 20 items, α = 0.92) and a tendency to experience anxiety (trait, 20 items, α = 0.90); Zung Self-Rating Depression Scale (46) assessing depression symptomatology during the previous week (20 items, α = 0.90); Athens Insomnia Scale (47) detecting sleeping disturbances in the last 7 days (7 items, α = 0.71); Functional Assessment of Chronic Illness Therapy-Fatigue Scale (48) measuring levels of fatigue the last 7 days (13 items, α = 0.94).

### Statistical Analysis

Comparisons for continuous and categorical variables were performed using the Mann–Whitney *U*-test and the Chi-square, respectively. To explore the perceived impact of negative stressful life events (defined as a negative stressful life event score > 3) in AAV development compared to RA and HC, odds ratios with 95% confidence intervals [ORs (95% CI)] were calculated in both univariate and multivariate models (stepwise backwards). Due to the relatively small sample size, only variables found to statistically differ at a level <0.01 in the univariate analysis were entered in the final models. Data analysis was performed using SPSS 23. Cronbach’s alpha (α) were calculated using SPSS.

## Results

### Demographics and Disease Characteristics

Demographic variables, disease characteristics, smoking, and alcohol habits are presented in Table S1 in Supplementary Material. Among AAV patients, mean age was 54.3 (SD 15.8) years, disease duration was 10.9 (SD 5.9) years and 56.6% were males. With respect to the clinical phenotype of AAV, 62.7% were classified as granulomatosis with polyangiitis (GPA), whereas 35.3% as microscopic polyangiitis. Disease activity, defined as BVAS > 0, was detected in 20.4% of the patients at the time of the interview.

### Negative Stressful Life Events, Coping Strategies, and Social Support

Table 1 displays the number and impact of stressful life events, as well as the developed coping strategies and available social support measures, 1 year prior to disease onset for AAV and RA patients and 1 year prior to the study entry for HC. Thirty-nine out of 53 (73.6%) AAV patients reported at least one negative stressful event prior to disease onset compared to 46 out of 85 (54.1%) RA patients and 32 out of 53 (60.4%) HC. While AAV patients and HC did not differ in the number of events reported, RA patients reported a significantly lower number of events prior to disease onset compared to both groups. Of note, 32.1% of AAV patients reported an event scoring >3 (range 0–10) compared to 13.2 and 11.8% in HC and RA, respectively (*p*-values: 0.035 and 0.004). The corresponding ORs and 95% CI between AAV patients as compared to HC and AAV as compared to RA controls are 3.1 (1.2–8.3), and 3.5 (1.5–8.5) and displayed in Table 3. Gender, ANCA type, clinical phenotype, BVAS at diagnosis did not affect the number or impact of stressful events (data not shown).

**Table 1:** Stressful life events, coping strategies, and social support*.

	AAV (*n* = 53)	HC (*n* = 53)	RA (*n* = 85)	*p*	*p*	*p*
				AAV vs HC	AAV vs RA	RA vs HC
**Negative life events, *n* (%)**						
No	14 (26.4)	21 (39.6)	39 (45.9)	0.21	0.030	0.48
Yes	39 (73.6)	32 (60.4)	46 (54.1)	0.21	0.030	0.48

**Negative life events**						
Number	1.2 ± 1.2	1.0 ± 1.15	0.7 ± 0.8	0.52	0.008	0.20
Scoring	2.8 ± 3.06	1.7 ± 2.3	1.8 ± 2.1	0.053	0.047	0.99

**Negative life events, *n* (%)**						
Number >2	8 (13.2)	7 (15.1)	5 (5.9)	0.50	0.081	0.21
Scoring >3	17 (32.1)	7 (13.2)	10 (11.8)	0.035	0.004	0.79

**COPE**						
Positive reinterpretation	9.2 ± 3.7	9.3 ± 3.4	9.2 ± 3.5	0.69	0.94	0.47
Mental disengagement	7.5 ± 2.4	6.8 ± 3.6	8.0 ± 2.4	0.039	0.23	0.003
Venting of emotions	9.4 ± 2.8	6.6 ± 3.2	10.5 ± 2.8	<0.001	0.033	<0.001
Use of social support	8.1 ± 3.7	8.9 ± 3.9	9.3 ± 3.5	0.20	0.038	0.55
Active	11.1 ± 3.4	9.5 ± 4.2	11 ± 3.1	0.037	0.72	0.032
Denial	7.1 ± 2.9	5.7 ± 3.1	7.0 ± 3.2	0.001	0.65	0.001
Religious	9.8 ± 4.9	6.3 ± 3.6	10 ± 4.9	<0.001	0.37	<0.001
Humor	7.6 ± 4.2	6.7 ± 3.4	8.1 ± 4.4	0.44	0.61	0.12
Behavioral disengagement	6.1 ± 2.7	6.0 ± 3.5	6.1 ± 3.0	0.33	0.66	0.45
Restraint	8.5 ± 2.6	9.4 ± 3.3	8.2 ± 2.8	0.077	0.62	0.026
Use of emotional support	8.6 ± 4.3	8.8 ± 3.6	9.8 ± 4.1	0.52	0.12	0.21
Substance use	4.4 ± 1.8	4.3 ± 1.5	4.4 ± 1.8	0.80	0.59	0.76
Acceptance	12.4 ± 3.2	9.1 ± 3.3	12.8 ± 3.1	<0.001	0.41	<0.001
Suppression of activities	9.4 ± 3.3	9.3 ± 4.4	9.2 ± 2.6	0.86	0.81	0.94
Planning	10.6 ± 3.3	10.1 ± 4.1	11.1 ± 3.1	0.69	0.30	0.19

**MOS-SSS**						
Emotional support	23.6 ± 7.9	25.2 ± 6.5	25.6 ± 10.3	0.64	0.24	0.66
Tangible support	14.2 ± 2.9	13 ± 3.7	14.3 ± 5.3	0.003	0.18	0.009
Affectionate support	10.7 ± 1.9	10.1 ± 2.7	11.08 ± 3.4	0.029	0.30	0.029
Positive social interaction	10.1 ± 2.5	11.7 ± 3.9	14.1 ± 4.9	0.25	<0.001	0.001
Total support	62.0 ± 13.3	62.0 ± 13.5	65.2 ± 21.4	0.84	0.33	0.17

*Values are mean ± SD unless indicated otherwise.

*AAV, ANCA-associated vasculitis; HC, healthy controls; RA, rheumatoid arthritis; COPE, COPE inventory; MOS-SSS, medical outcomes study, social support survey*.

As far as the coping strategies developed, AAV patients scored higher than HC in active coping (efforts to remove a stressor) and acceptance (accepting that the stressor has occurred) and religious coping (engagement in religious activities). They also scored higher than HC in mental disengagement (psychological distraction from the stressor), venting of emotions (discharging negative feelings) and denial (not accepting a situation). Whereas AAV patients and HC did not differ in the use of social support as a coping mechanism, RA patients were found to use more social support than both groups, a difference that was found to be statistically significant, when compared to AAV patients (*p*-value: 0.038).

In regard to available support resources, AAV patients reported more tangible (the provision of material aid or behavioral assistance), and affectionate support (the expressions of love and affection) than HC (*p*-value: 0.003 and *p*-value: 0.034, respectively), but less positive social interaction as compared to RA (*p*-value: <0.001). The latter also reported greater social interaction even compared to HC (*p*-value: 0.001). Total social support index did not differ between groups.

### Psychological Variables

In Table 2, psychological profiles of AAV patients and controls are presented. AAV patients compared to RA but not HC group were more likely to exhibit psychoticism >2 [ORs (95% CI): 2.6 (1.2–5.8), *p*-value: 0.025]. Moreover, RA patients demonstrated lower psychoticism rates compared to HC group [ORs (95% CI): 0.2 (0.1–0.5), *p*-value: <0.001]. No other differences with respect to psychological dimensions examined, emerged between groups.

**Table 2:** Odds ratios of psychometric variables between AAV patients and controls.^a^

	AAV vs HC	*p*	AAV vs RA	*p*	RA vs HC	*p*
	OR (95% CI)		OR (95% CI)		OR (95% CI)	
ZSDS >40	0.8 (0.3–2.4)	0.77	1.3 (0.6–3.0)	0.54	0.6 (0.2–1.8)	0.37
STAI state >35	1.1 (0.3–3.3)	0.99	1.1 (0.5–2.3)	0.99	0.9 (0.3–2.9)	0.99
STAI trait >35	0.4 (0.1–1.3)	0.17	0.8 (0.4–1.6)	0.59	0.5 (0.2–1.2)	0.30
EPQ N >12	0.4 (0.2–1.3)	0.16	0.8 (0.4–1.6)	0.57	0.6 (0.2–1.6)	0.30
EPQ P >2	0.4 (0.1–1.2)	0.11	2.6 (1.2–5.8)	0.025	0.2 (0.1–0.5)	<0.001
EPQ E >9	0.6 (0.2–2.3)	0.53	1.3 (0.6–2.8)	0.56	0.5 (0.1–1.7)	0.26
AIS >6	1.4 (0.4–4.8)	0.76	1.0 (0.5–2.1)	0.99	1.4 (0.4–4.6)	0.77
FACIT-F >30	1.4 (0.4–4.8)	0.76	1.8 (0.8–4.2)	0.19	1.2 (0.3–3.9)	0.75

*^a^Cut-off scores are presented as shown in previous study (31)*.

*AAV, ANCA-associated vasculitis; HC, healthy controls; RA, rheumatoid arthritis; OR, odds ratio; 95% CI = 95% confidence interval; ZSDS, Zung Self-Rating Depression Scale; STAI, State and Trait Anxiety Inventory; EPQ N, Eysenck Personality Questionnaire, Neuroticism; EPQ P, Eysenck Personality Questionnaire, Psychoticism; EPQ E, Eysenck Personality Questionnaire, Extraversion; AIS, Athens Insomnia Scale; FACIT-F, Functional Assessment of Chronic Illness Therapy-Fatigue Scale*.

### Multivariate Analysis

We next wished to explore whether the association between the perceived impact of negative stressful life events (defined as scoring > 3) and AAV development compared to HC and RA remained significant, after other contributing psychosocial variables—found to differ at level < 0.01 in univariate comparisons between groups—were taken into account (Table 3). Indeed, an increased risk for AAV development in patients reporting scoring of stressful events >3 in relation to HC and RA controls was demonstrated [ORs (95% CI): 4.4 (1.0–19.0) and 4.6 (1.6–13.4), respectively] (Table 3).

**Table 3:** Increased risk for AAV, in patients experiencing stressful event scores >3, 1 year prior to disease onset.

Scoring of stressful life events more than 3			
AAV vs HC		AAV vs RA	
OR (95% CI)		OR (95% CI)	
Univariate analysis	Multivariate analysis^a^	Univariate analysis	Multivariate analysis^b^
3.1 (1.2–8.3)	4.4 (1.0–19.0)	3.5 (1.5–8.5)	4.6 (1.6–13.4)

*^a^Adjusted for education, venting of emotions, acceptance, religious, denial (coping), tangible support (social support) stressful life events scoring >3 (all variables less than 0.01)*.

*^b^Adjusted for sex, disease duration, number of stressful life events, positive social interaction, psychoticism, stressful life events scoring >3 (all variables less than 0.01)*.

*AAV, ANCA-associated vasculitis; HC, healthy controls; RA, rheumatoid arthritis; OR, odds ratio; 95% CI, 95% confidence interval*.

## Discussion

To the best of our knowledge, this is the first report so far, revealing an independent association of subjective perception of psychological stress (defined as the scoring of negative stressful life events >3) prior to disease onset and AAV development, after other psychosocial variables, previously thought to be involved in stress handling, were considered. While AAV patients and HC did not differ in the number of events reported, RA patients reported a significantly lower number of events prior to disease onset compared to both groups. In regard to coping strategies and available social support in the presence of a stressor, AAV patients compared to HC were found to use more frequently mental disengagement, venting of emotions, denial and turning to religion, as well as active coping and acceptance and they tended to receive more tangible and affectionate support. With the exception of less venting of emotions, use of social support and positive social interaction, AAV patients reported similar trends in the majority of stress management strategies implemented, in comparison to the RA group, as expected due to the chronic nature of both diseases. As far as personality characteristics, only psychoticism trait was found to occur more frequently among AAV patients compared to RA group. No differences in depression, anxiety, fatigue, or sleep disturbances scorings between patients and controls were detected.

The main finding from the current analysis was that the perceived degree of negative impact of stressful life events was independently associated with AAV status, even after adjusting for potential confounding psychosocial variables. This observation was previously shown as a trigger of disease development or disease flare-ups in other autoimmune diseases such as SS (8), SLE (29), Grave’s disease (6, 7), SSc (32), childhood arthritis (9), and RA (35). The link between mental health and increased likelihood of disease flare ups has also been suggested in patients with Wegener’s granulomatosis (28). Although the number of reported negative life events has been implicated in disease onset in other autoimmune disorders (6–9), the lack of such association in the present study might be related to the limited number of sample size, as a result of AAV rarity. The lower number and impact of stressful events in RA compared to AAV patients might be attributed to the different male to female ratio between the two groups, with more males in the AAV group. According to previously formulated hypotheses, women tend to attribute less their disease onset to a stressing event (5), while men are considered to be more biologically and psychosocially vulnerable (49).

Potential underlying mechanisms between chronic stress and disease development include undesirable behavioral changes (such as smoking, absence of exercise, excessive alcohol consumption, poor diet habits) and/or involvement of hypothalamic–pituitary–adrenal (HPA) axis and the sympathetic adrenal medullary (SAM) system (50). While following an acute stressor, cortisol is released, chronic stressors have been related to suppression of HPA axis (51) leading to reduced production of endogenous anti-inflammatory glucocorticoids fueling a systemic inflammatory response. On the other hand, activation of SAM leads to catecholamine release, which in combination with the autonomous nervous system can affect immune functions (50), promoting potentially disease development.

As a buffer to the excessive environmental pressures, coping strategies and received social support are implemented to ensure a better adaptation and resolution of intense emotions. For instance, spouse support in RA patients and relational mutuality have been found to relate to patient’s psychological well being (52, 53). In the present report, AAV patients tend to implement both active (such as active coping, acceptance, religious) and avoidant strategies (mental disengagement, venting of emotions, denial), as well as to receive more tangible and affectionate support. In a previous study, avoidant or dysfunctional coping styles—previously shown to be implicated in non-expression and non-processing of emotions related to stressors, leaving them unresolved (54)—have been shown to contribute to poor quality of life in AAV patients (27) and to predict psychological distress and increased disease activity in other autoimmune diseases (8, 29, 32, 34). Higher levels of support, mentioned by AAV patients, might reflect their need to receive attention, when encountering a stressful situation. The scale implemented in the present study, reveals the provision and availability of aspects of support perceived by patients, not the identification of the sources of support, neither an assertive strategy. However, it should be emphasized that the differences in coping strategies and social support reported between AAV patients and controls could reflect the effect of chronic disease, though the relevant questionnaires on coping strategies and social support were addressed for the period prior to disease onset as a buffer to the stressful events mentioned.

Finally, it was found that AAV patients had lower scores than HC—though non significant—in the psychoticism dimension of the personality questionnaire. This trend may be associated with higher religiosity reported by AAV participants, in line with previous observations (55). Of note, patients and controls presented with relatively low mean scores (2.2 and 3.8, respectively), indicating the absence of true psychotic features. RA controls presented with significantly lower scores than AAV patients and this may also be attributed to the higher religiosity reported by this group. Religiosity as a coping strategy was found to negatively correlate to psychoticism dimension, in our group samples (rho: 0.219, *p*-value: 0.006) (data not shown), validating the above interpretation.

Interestingly, no differences in depression, anxiety, fatigue, or sleep disturbances scorings between patients and controls were found. Previous work by Basu et al. (26, 27) found higher overall psychological distress in AAV patients, associated to poor physical and mental quality of life in those patients. Similarly, worse mental health has been linked to increased disease flares in those patients (28). A number of other observations have reported higher depression and anxiety and overall increased psychological distress in patients with related conditions (29, 31, 32). Yet, this was not confirmed in our results, probably revealing a well-regulated cohort.

There is a number of limitations in the current study starting with the small sample size and the subjectivity of the self-reported measurements. Of the strength points, we were very cautious in our data collection concerning the timing of the stressful life event, matching the chronological period of time of the reported events in patients and controls. Undoubtedly, prospective studies using large-scale population registries would be highly desirable to validate our findings.

Taken together, the current findings support early institution of psychological therapies as a complementary tool for disease management among AAV individuals. Strategies implemented in the stress and emotional modification, include patient education, behavioral therapies, mindfulness-based therapies, and cognitive behavioral therapies (CBT) (56, 57) The latter—widely used in autoimmune populations (58)—aim at stress management through coping strategies and social skills training, emotion regulation as well as cognitive restructuring of negative thoughts about pain. While several studies in RA patients revealed a long-term efficacious role of CBT interventions in pain behavior, disease activity, joint stiffness, anxiety and depression, others did not confirm the positive effects on disease activity or joint counts, though they did maintain positive outcomes on anxiety, depression, fatigue, and pain management (10); inhibition of IL-6 production seems to mediate the observed positive effects (59). In conclusion, we present for the first time evidence of the impact of stress in AAV onset, as previously shown in other autoimmune disorders, implying a crucial role of psychological interventions along with medical treatment in AAV management.

## Ethics Statement

This study was carried out in accordance with the recommendations of “Laiko General Hospital Ethics Committee” with written informed consent from all subjects. All subjects gave written informed consent in accordance with the Declaration of Helsinki. The protocol was approved by the “Laiko General Hospital Ethics Committee.”

## Author Contributions

CG, CM, and HM conceived and designed the work. CG, CM, DK, SL, and HM contributed in the recruitment of participants and the acquisition and interpretation of data. CG, CM, and SL analyzed and reviewed the work. CG, CM, SL, DK, and HM reviewed and approved the final version of the study.

## Conflict of Interest Statement

The authors declare that the research was conducted in the absence of any commercial or financial relationships that could be construed as a potential conflict of interest.
