# Supplementary material for: Stress and Disease Onset in Antineutrophil Cytoplasmic Antibody-Associated Vasculitis
Source: Front Psychiatry. 2017 Dec 15;8:286. doi: 10.3389/fpsyt.2017.00286 (PMC5770743; doi:10.3389/fpsyt.2017.00286)
Supplement: Supplementary file 1 [file Data_Sheet_1.DOCX]

Supplementary Material

**Stress and Disease onset in ANCA-Associated Vasculitis**

Christina Golemati^1*^ PhD, Clio P. Mavragani^1,2^  MD, Sophia Lionaki ^3^ MD, Dimitrios Karaiskos^1^ MD, Haralampos M. Moutsopoulos^1^ MD, FACP, FRCP (hc)

^1^Department of Pathophysiology, Faculty of Medicine, National and Kapodistrian University of Athens, Athens, Greece

^2^Department of Physiology, Faculty of Medicine, National and Kapodistrian University of Athens, Athens, Greece

^3^Department of Nephrology, Laiko Hospital, National and Kapodistrian University of Athens, Athens, Greece

Correspondence:

*Christina Golemati

[chrisgolemati@yahoo.gr](mailto:chrisgolemati@yahoo.gr)

| **Supplementary table 1**: Demographic variables and disease characteristics* | | | | | | |
| --- | --- | --- | --- | --- | --- | --- |
|  | **AAV**  **(n=53)** | **HC**  **(n=53)** | **RA**  **(n=85)** | ***P***  **AAV vs**  **HC** | ***P***  **AAV**  **vs**  **RA** | ***P***  **RA**  **vs**  **HC** |
| Men, women | 30 (56.6),  23 (43.4) | 30 (56.6), 23 (43.4) | 15 (17.6), 70 (82.4) | 0.58 | *<0.001* | *<0.001* |
| Age (years), mean± SD | 54.3±15.8 | 53.2±12.3 | 56.2±15.0 | 0.44 | 0.37 | 0.13 |
| Disease duration (years), mean± SD | 10.9 ±5.9 | NA | 16.9 ±12.2 | ΝΑ | *0.008* | NA |
| BVAS^¶^, mean± SD  BVAS^¶^>0  BVAS at dx, mean± SD | 1.8 ±4.9  10 (20.4)  16±6.4 | NA | NA | NA | NA | NA |
| ANCA type†  PR3/C-ANCA  MPO/P-ANCA | 37 (71.2)  15 (28.8) | ΝΑ | ΝΑ | ΝΑ | ΝΑ | ΝΑ |
| Clinical Phenotype‡  MPA  GPA | 18 (35.3)  32 (62.7) | ΝΑ | ΝΑ | ΝΑ | ΝΑ | ΝΑ |
| Personal status  Married  Widowed  Divorced  Separated  In Relationship  Single | 35 (66.0)  5 (9.4)  3 (5.7)  5 (9.4)  5 (9.4)  0 (0.0) | 43 (81.1)  0 (0.0)  4 (7.5)  4 (7.5)  2 (3.8)  0 (0.0) | 58 (68.2)  6 (7.1)  3 (3.5)  2 (2.4)  7 (8.2)  9 (10.6) | 0.061  *0.028*  0.50  0.50  0.22  - | 0.85  0.75  0.67  0.11  >.99  *0.013* | 0.11  0.082  0.42  0.20  0.48  *0.013* |
| Place of residence  Big city  Suburbs  Small town  Village | 35 (66.0)  5 (9.4)  12 (22.6)  1 (1.9) | 38 (71.7)  9 (17)  5 (9.4)  1 (1.9) | 58 (68.2)  7 (8.2)  17 (20)  3 (3.5) | 0.34  0.19  *0.055*  0.75 | 0.85  >.99  0.83  >.99 | 0.70  0.17  0.15  >.99 |
| Educational status  <primary school  Primary school  Secondary school  High school  College  University  Postgraduate | 2 (3.8)  4 (7.5)  4 (7.5)  13 (24.5)  11 (20.8)  12 (22.6)  7 (13.2) | 1 (1.9)  7 (13.2)  17 (32.1)  14 (26.4)  4 (7.5)  6 (11.3)  4 (7.5) | 2 (2.4)  13 (15.3)  5 (5.9)  23 (27.1)  22 (25.9)  13 (15.3)  7 (8.2) | 0.50  0.26  *0.001*  0.50  *0.046*  0.098  0.26 | 0.63  0.28  0.73  0.84  0.54  0.36  0.39 | >.99  0.80  <0.001  >.99  *0.007*  0.61  >.99 |
| Smoking  No  Yes  Quit | 36 (67.9)  5 (9.4)  12 (22.6) | 26 (49.1)  16 (30.2)  11 (20.8) | 49 (57.6)  17 (20)  19 (22.4) | 0.076  0.013  >.99 | 0.28  0.15  >.99 | 0.21  0.38  >.99 |
| Alcohol  >14 units/week | 5 (9.4) | 4 (7.5) | 1 (1.2) | >.99 | *0.031* | 0.072 |
| *Values are the number (percentage) unless indicated otherwise  **^¶^**BVAS at time of the interview  †n=52  ‡n=51  AAV = ANCA-associated vasculitis; HC = healthy controls; RA = rheumatoid arthritis; NA = non-applicable; BVAS = Birmingham Vasculitis Activity Scale; PR3 = proteinase 3; MPO = myeloperoxidase; MPA = microscopic polyangiitis; GPA = granulomatosis polyangiitis; | | | | | | |
